# A Tale of Two Surges: Differences in Outcomes in the COVID-19 Pandemic in a Community Teaching Hospital in Massachusetts

**DOI:** 10.7759/cureus.21547

**Published:** 2022-01-24

**Authors:** Gabriela C Milla-Godoy, Klaorat Prasongdee, Cagney Cristancho, Alekya Poloju, Felipe Barbosa, Thomas Treadwell

**Affiliations:** 1 Internal Medicine, MetroWest Medical Center, Tufts University School of Medicine, Framingham, USA; 2 Infectious Disease, MetroWest Medical Center, Tufts University School of Medicine, Framingham, USA

**Keywords:** covid-19, covid-19 cases, covid-19 mortality, retrospective research, infectious disease medicine

## Abstract

Background

The coronavirus disease 2019 (COVID-19) pandemic has challenged the scientific community in the prompt implementation of therapies. We report and contrast characteristics and outcomes from two COVID-19 surges in March 2020 and December 2020 in patients at MetroWest Medical Center in Framingham.

Methods

The study was conducted at MetroWest Medical Center. We extracted the data of 315 patients from March 17, 2020, to June 30, 2020, and 104 patients from November 19, 2020, to December 30, 2020. All patients were inpatients and had confirmed severe acute respiratory syndrome coronavirus 2 (SARS-CoV-2) infection by polymerase chain reaction (PCR). We extracted the patient’s demographic information, clinical data, and given treatments. We also examined comorbidities and categorized them by the Charlson Comorbidity Index (CCI). The primary endpoints were intensive care unit (ICU) level of care, mechanical ventilation, or death.

Results

A total of 419 patients were studied. The median age was 76. During the first surge (S1), 150 (47%) were from nursing homes and 133 (42%) were from independent living. More than half (72) of the independent living patients had a primary language other than English. During the second surge (S2), 12% (13) were from nursing homes. The most common comorbidities were similar for both groups and included obesity, diabetes, and chronic lung disease. However, during the first surge, 33% (104) of the patients had dementia. The median Charlson Comorbidity Index score was worse in the first surge; the predicted 10-year survival was 21% versus 53%. The treatments given included remdesivir in 5% (16) in the first surge versus 60% (62) in the second surge. Dexamethasone was given only in the second surge in 69% (72) of the patients.

Outcomes

The reported outcomes are contrasted by the first versus the second surge. Admission to the intensive care unit was required in 83 (27%) of the patients during the first surge versus 15 (14%) of the patients during the second surge. Mechanical ventilation was required in 33 (11%) of the patients during the first surge versus 5 (11%) of the patients during the second surge. The overall mortality was 25% during the first surge (79) versus 9% (9) during the second surge.

Conclusion

Among patients with COVID-19 infection admitted to a community teaching hospital during the second Massachusetts surge, there was a significant improvement in clinical outcomes, particularly mortality, compared with patients admitted during the early pandemic. It is tempting to attribute the improved outcomes to the implementation of treatment with corticosteroids and more use of antiviral therapy. However, the patients admitted during the larger first surge were more likely to have a do not resuscitate (DNR) status on admission, be from a nursing home, have dementia, and have poorer predicted survival.

## Introduction

The first cases of novel coronavirus disease 2019 (COVID-19) were confirmed in late January 2020 in the state of Washington [1]. By early March, COVID-19 had spread across the United States, most heavily affecting urban areas such as New York City [2]. The first surge (S1) of cases occurred in Massachusetts at that time [3]. The MetroWest Medical Center is a community teaching hospital with two campuses in Natick and Framingham, Massachusetts. This suburban area west of Boston has many nursing homes, skilled nursing facilities (SNFs), and group homes, patients from which were severely affected during the pandemic [4,5]. In addition, our community has an emerging immigrant population from Latin America and sub-Saharan Africa. We report the demographics, clinical manifestations, treatment, and outcomes for the first 315 cases of COVID-19 infection at our hospital from the middle of March to late June 2020. We then gathered data on a second and smaller group of patients (104 cases) who were admitted during a second surge (S2) in December 2020.

This article was previously presented as a research abstract electronic poster presentation for the American College of Physicians Massachusetts Chapter Meeting on Saturday, October 16, 2021.

## Materials and methods

The study was conducted at both campuses of the MetroWest Medical Center, located in Framingham and Natick, Massachusetts. We extracted the data of 315 patients from March 17, 2020, to June 30, 2020 (S1), following which we gathered data on 103 patients admitted in December 2020 (S2). Written consent was waived in light of the need to collect data in the setting of a public health emergency. All patients were in the inpatient setting either wards or intensive care unit and had confirmed severe acute respiratory syndrome coronavirus 2 (SARS-CoV-2) infection by a positive reverse-transcriptase polymerase chain reaction (RT-PCR) obtained by nasopharyngeal sample. The results were confirmed in our facility, or patients came from outside of the facility with a confirmatory test. The clinical outcomes for the S1 group were monitored until June 30, 2020, the data cutoff. For patients who were readmitted during the study period, the data regarding the first admission are presented, and a separate readmission outcome is described.

Data were collected using the hospital’s electronic medical record (Meditech, Medical Information Technology, Inc., MA, USA). A reporting database was used by all authors. Continuous variables were expressed as means and medians, and categorical variables were expressed in counts, percentages, and ranges. Data were analyzed and interpreted by the authors using R programming language report version 4.0.4 [6].

We extracted the patient’s demographic information, including age, sex, race, primary and preferred language, living situation (including independent living, nursing home, assisted living facility, group homes, or prison), and occupation. Clinical data included the body mass index (BMI), days on a ventilator and high flow, code status upon admission, and laboratory values (including ferritin, C-reactive protein (CRP), procalcitonin, creatinine phosphokinase, troponin, and D-dimer levels). The treatments included were convalescent plasma, ceftriaxone, azithromycin, doxycycline or other antibiotics prior to admission, hydroxychloroquine, tocilizumab, and remdesivir. During S1, corticosteroids were not recommended, but we recorded steroid use during S2. We also examined comorbidities including diabetes, congestive heart failure, chronic lung disease, immunosuppression, chronic kidney disease, dementia, and liver disease. The clinical outcomes included discharge disposition and death. The primary endpoints were ICU level of care, mechanical ventilation, or death. All outcomes available, such as ICU level of care, mechanical ventilation, or death, were completed by the end of the study date. We used the Charlson Comorbidity Index (CCI), a method of categorizing comorbidities of patients based on their diagnosis [7]. Chronic kidney disease was defined as the presence of either kidney damage or decreased kidney function with a glomerular filtration rate of <60 mL/minute/1.73 m^2^ for three or more months irrespective of the cause or functional abnormalities according to the Kidney Disease: Improving Global Outcomes (KDIGO) definition [8]. Chronic lung disease included the spectrum of chronic obstructive lung disease, emphysema, and chronic obstructive asthma [9].

## Results

The demographic characteristics of the patients are shown in Table 1.

**Table 1:** General characteristics of the patients hospitalized with COVID-19

Demographics	S1 (number (%))	S2 (number (%))
Total	315	104
Age (median (range))	73 (20–105)	76 (21–103)
Sex		
Male	170 (54%)	54 (52%)
Female	145 (46%)	50 (48%)
Race		
White	228 (72%)	80 (77%)
Other	54 (17%)	16 (16%)
African American	21 (7%)	6 (6%)
Asian	8 (2%)	1 (1%)
Unknown	4 (1%)	1 (1%)
Primary language		
English	229 (73%)	75 (72%)
Spanish	41 (13%)	18 (17%)
Other	18 (6%)	3 (3%)
Portuguese	17 (5%)	7 (7%)
Missing	7 (2%)	3 (3%)
Arabic	2 (0.6%)	1 (1%)
French creole	1 (0.3%)	0 (0%)
Unknown	5 (2%)	0 (0%)
Healthcare worker		
Yes	36 (11%)	3 (3%)
No	230 (73%)	99 (95%)
Unknown	41 (16%)	0 (0%)
Living situation		
Nursing home/skilled nursing facility	150 (47%)	13 (12%)
Home	133 (42%)	78 (75%)
Assisted/independent living	15 (5%)	11 (11%)
Group home	9 (3%)	2 (2%)
Prison	6 (2%)	0 (0%)
Missing	2 (0.6%)	0 (0%)
Facility		
Framingham Union Hospital	256 (81%)	104 (100%)
Leonard Morse Hospital	59 (19%)	0 (0%)
Body mass index (kg/m^2^)	26.8 (14–70.6)	28.1 (14.9–323)
Do not resuscitate at admission		
Yes	107 (34%)	89 (86%)
No	207 (65%)	15 (14%)
Unknown	1 (0.3%)	
Diabetes		
Yes	78 (25%)	32 (31%)
No	200 (64%)	71 (69%)
Missing	37 (12%)	1 (1%)
Chronic lung disease		
Yes	59 (19%)	20 (19%)
No	210 (67%)	84 (81%)
Missing	46 (14.6%)	
Congestive heart failure		
Yes	49 (16%)	20 (19%)
No	225 (71%)	84 (81%)
Missing	41 (13%)	
Chronic kidney disease		
Yes	57 (18%)	20 (19%)
No	219 (70%)	84 (81)
Missing	39 (12%)	
Severe psychiatric disease		
Yes	35 (11%)	8 (7%)
No	190 (60%)	96 (92%)
Missing	90 (27%)	
Dementia		
Yes	104 (33%)	8 (8%)
No	177 (56%)	96 (92%)
Missing	34 (11%)	0 (0%)
Charlson Comorbidity Index(median (range))	5 (0–12)	4 (0–11)
Charlson 10-year probability of survival	21%	53%

During the first surge (S1), a total of 315 patients were studied, 256 (81%) at Framingham Union campus and 59 (19%) at Leonard Morse campus. The median age was 73 (range: 20-105), 170 (54%) were male, and 228 (72%) were White. Most patients presented from conglomerate settings, including 150 (47%) from nursing homes or skilled nursing facilities. The most common primary language was English (229, 73%); however, in the subgroup of patients living in non-congregate settings, half 72 (55%) had a primary language other than English. Thirty-six (11%) of the patients were healthcare workers.

The median BMI was 26.8 kg/m^2^ (range: 14-71 kg/m^2^). At admission, 34% of the patients had a do not resuscitate (DNR) code status. The most common comorbidities were dementia (33%), diabetes (25%), obesity (24%), and chronic lung disease (19%). The baseline characteristics of the patients in S2 were quite similar, except that far fewer patients were from nursing homes or SNFs and there were fewer patients with DNR status and dementia.

The median score on the Charlson Comorbidity Index (CCI) was 5 (range: 0-12), corresponding to a 21% estimated 10-year survival. The estimated 10-year survival for patients during S2 was 53%.

The laboratory findings are presented in Table 2. Most patients had elevated levels of ferritin (median: 549 ng/mL; range: 34-41,400) and CRP (median: 103 mg/L; range: 1.4-3460). The mean D-dimer was 6.19 μg/mL (SD: 24.9). The median procalcitonin was 0.18 ng/mL (range: 0.03-26.1).

**Table 2:** Laboratory results of patients hospitalized with COVID-19

Laboratories	S1 (median (range))	S2 (median (range))	Reference range
Ferritin peak (ng/mL)	594 (34–41,400)	584 (43–8000)	30–400
C-reactive protein peak (mg/dL)	103 (1.4–3460)	49 (0.6–331)	0–5
Procalcitonin (ng/mL)	0.185 (0.03–26.1)	0.150 (0.04–2.3)	0.00–0.08
D-dimer peak (μg/mL)	Mean: 6.19 (SD: 24.9)	Mean: 3.30 (SD: 5.63)	0.00–0.49

The treatments are presented in Table 3 and included ceftriaxone (73%), doxycycline (48.3%), azithromycin (30.8%), hydroxychloroquine (26.3%), convalescent plasma (3.2%), tocilizumab (6.3%), and remdesivir (5.1%). A total of 124 (39.4%) patients received antibiotics prior to hospital admission. During S2, fewer patients received antibiotics (34, 33%), and 60% of the patients received remdesivir. Seventy-two (69%) patients received corticosteroids during the second surge.

**Table 3:** Treatments of patients hospitalized with COVID-19

Treatments	S1 (median (%)) (N = 315)	S2 (median (%)) (N = 104)
Antibiotics received prior to admission	124 (39%)	34 (33%)
Ceftriaxone	231 (73%)	42 (40%)
Azithromycin	97 (31%)	12 (12%)
Doxycycline	152 (48%)	20 (19%)
Hydroxychloroquine	83 (26%)	0 (0%)
Convalescent plasma	10 (3%)	0 (0%)
Tocilizumab	20 (6%)	0 (0%)
Remdesivir	16 (5%)	62 (60%)
Dexamethasone	N/A	72 (69%)

Outcomes

During the first surge, admission to the intensive care unit was required in 86 (27%) of the patients. High-flow nasal cannula oxygen was delivered in 29 (9%), with a median of three days (range: 1-15). Mechanical ventilation was required in 33 (11%), with a median of 10 days (range: 1-76). The overall mortality was 25% (79) (Table 4).

For patients who survived, 107 (34%) of patients were discharged either to nursing homes or rehabilitation facilities, and 121 (38%) were discharged home (Table 4). Of the 315 patients, 20 (6%) were readmitted for all causes, and six (2%) of them expired.

**Table 4:** First surge outcomes by 10-year age intervals of patients hospitalized with COVID-19 COVID-19: coronavirus disease 2019; ICU: intensive care unit; LTC: long-term care including short-term rehabilitation, long-term acute care, nursing home, and skilled nursing facilities

Age intervals	ICU level of care	Mechanical ventilation	Discharged home	Discharged LTC	Death
Years	Number (%)	Number (%)	Number (%)	Number (%)	Number (%)
<40	1 (3%)	0 (0%)	22 (79%)	3 (11%)	0 (0%)
40–49	2 (8%)	1 (4%)	22 (92%)	1 (4%)	1 (4%)
50–59	14 (48%)	7 (24%)	18 (62%)	6 (21%)	12 (20%)
60–69	18/59 (31%)	11/59 (17%)	21 (36%)	26 (44%)	0/59 (0%)
70–79	23 (37%)	9 (15%)	17 (27%)	23 (37%)	20 (32%)
>80	28 (25%)	5 (4%)	21 (19%)	48 (43%)	42 (37%)
Total	86 (27%)	33 (11%)	121 (38%)	107(34%)	79 (25%)

During the second surge, admission to the intensive care unit was required in 15 (14%) of the patients. High-flow nasal cannula oxygen was delivered in 15 (14%), with a median of four days (range: 1-18). Mechanical ventilation was required in 5 (4.8%), with a median of five days (range: 1-15). The overall mortality was 9 (8.7%). For patients who survived, 22 (21%) were discharged to nursing homes or rehabilitation facilities, and 73 (70%) were discharged to home (Table 5).

**Table 5:** Second surge outcomes by 10-year age intervals of patients hospitalized with COVID-19 COVID-19: coronavirus disease 2019; ICU: intensive care unit; LTC: long-term care including short-term rehabilitation, long-term acute care, nursing home, and skilled nursing facilities

Age intervals	Number	ICU level of care	Mechanical ventilation	Discharged home	Discharged LTC	Death
Years		Number (%)	Number (%)	Number (%)	Number (%)	Number (%)
<40	11	1 (9%)	1 (9%)	10 (91%)	1 (9%)	0 (0%)
40–49	0	0 (0%)	0 (0%)	0 (0%)	0 (0%)	0 (0%)
50–59	16	2 (13%)	1 (6%)	15 (94%)	1 (6%)	0 (0%)
60–69	12	3 (25%)	2 (17%)	9 (75%)	2 (17%)	1 (8%)
70–79	26	3 (12%)	0 (0%)	17 (65%)	8 (31%)	1 (4%)
>80	39	6 (15%)	1 (2%)	22 (56%)	10 (26%)	7 (18%)
Total	104	15 (14%)	5 (4.8%)	73 (70%)	22 (21%)	9 (9%)

Outcomes by age and comorbidities

The outcomes by 10-year age intervals are described in Table 4 andTable 5. During the first surge, for ICU level of care, the predominant age group was 80 years and above (28, 24.8%). For patients requiring mechanical ventilation, the most common age group was 60-69 years old (11, 17%). For patients who had a fatal outcome, 42 (37%) were 80 years or older (Table 4).

Table 6 presents the outcomes of COVID-19 patients with obesity (BMI ≥ 30 kg/m^2^). During the first surge, 23 (30.3%) required ICU level of care, 13 (17.1%) required mechanical ventilation, and 14 (18.4%) expired.

**Table 6:** Outcomes of COVID-19 patients with obesity COVID-19: coronavirus disease 2019; ICU: intensive care unit; LTC: long-term care including short-term rehabilitation, long-term acute care, nursing home, and skilled nursing facilities

Surge	Obesity	ICU level of care	Mechanical ventilation	Discharged home	Discharged LTC	Death
Number	Number	Number (%)	Number (%)	Number (%)	Number (%)	Number (%)
S1 (315)	76	23 (30%)	13 (17)	43 (56%)	18 (24%)	14 (19%)
S2 (104)	38	5 (13%)	3 (8%)	29 (77%)	8 (21%)	1 (3%)

**Figure 1:**
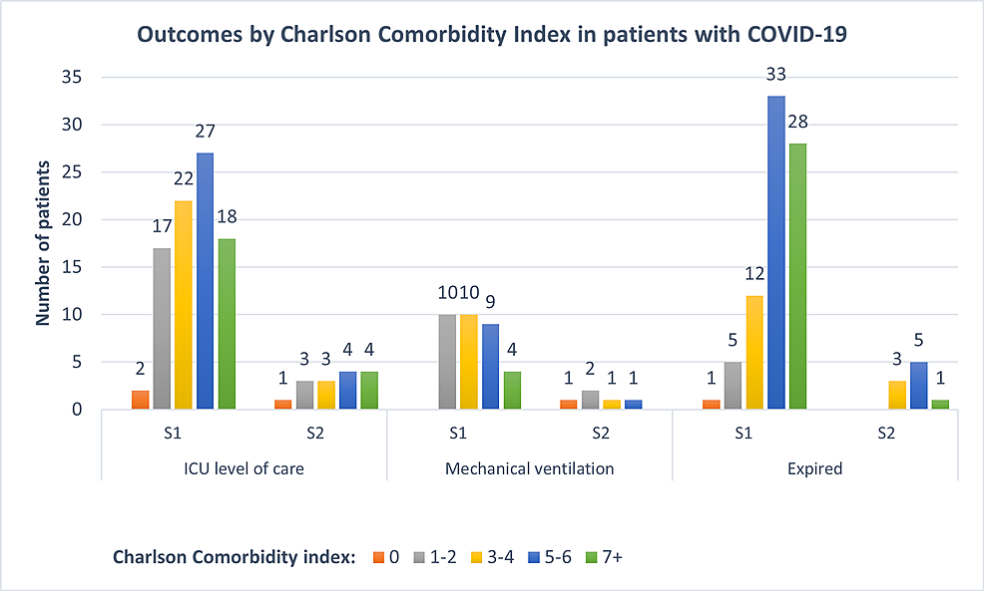
Outcomes by Charlson Comorbidity Index in patients with COVID-19

Outcomes were stratified according to the Charlson Comorbidity Index (CCI) and are depicted inTable 7 and Table 8. A comparative table by endpoints on both surges is presented in Figure 1. As expected, there was a clear-cut association between CCI and mortality, and nearly half of patients with a CCI of 7 or greater died.

**Table 7:** First surge outcomes by Charlson Comorbidity Index (CCI) COVID-19: coronavirus disease 2019; ICU: intensive care unit; LTC: long-term care including short-term rehabilitation, long-term acute care, nursing home, and skilled nursing facilities

CCI	Number	ICU level of care	Mechanical ventilation	Discharged home	Discharged LTC	Expired
		Number (%)	Number (%)	Number (%)	Number (%)	Number (%)
0	36	2 (6%)	0 (0%)	31 (86%)	2 (6%)	1 (3%)
1–2	53	17 (32%)	10 (19%)	38 (72%)	9 (17%)	5 (9%)
3–4	62	22 (35%)	10 (16%)	27 (43%)	22 (35%)	12 (19%)
5–6	105	27 (26%)	9 (9%)	18 (17%)	51 (49%)	33 (31%)
7+	58	18 (31%)	4 (7%)	7 (12%)	23 (40%)	28 (48%)
Total	315	86 (27%)	33 (10%)	121 (38%)	107 (34%)	79 (25%)

**Table 8:** Second surge outcomes by Charlson Comorbidity Index (CCI) COVID-19: coronavirus disease 2019; ICU: intensive care unit; LTC: long-term care including short-term rehabilitation, long-term acute care, nursing home, and skilled nursing facilities

CCI	Number	ICU level of care	Mechanical ventilation	Discharged home	Discharged LTC	Expired
		Number (%)	Number (%)	Number (%)	Number (%)	Number (%)
0	5	1 (20%)	1 (20%)	4 (80%)	1 (20%)	0 (0%)
1–2	24	3 (87%)	2 (8%)	23 (96%)	1 (4%)	0 (0%)
3–4	30	3 (10%)	1 (3%)	23 (77%)	4 (13)	3 (10%)
5–6	28	4 (14%)	1 (4%)	16 (57%)	7 (25%)	5 (18%)
7+	17	4 (23%)	0 (0%)	7 (41%)	9 (53%)	1 (6%)
Total	104	15 (14%)	5 (5%)	73 (70%)	22 (21%)	9 (8%)

## Discussion

Beginning in March 2020, the COVID-19 pandemic severely affected Massachusetts. During the first surge that peaked in late April, the number of diagnoses exceeded 2500 patients per day, and the peak hospitalization rate was approximately 4000 patients per day [10]. COVID-19 was particularly savage in nursing homes and extended care facilities [5]. Lack of training, poor availability of personal protective equipment, and overwhelming numbers of cases resulted in a rapid spread of infection, and many healthcare workers were also infected [11].

Beginning in November 2020, the second surge in Massachusetts began, peaking at more than 7500 cases per day in early January [10]. In contrast to the first surge, the percentage of positive tests, hospitalization rate, and death rate were all lower during the second surge. These trends have been noted in several other states.

The town of Framingham, which recently became incorporated as a city, has a rich tradition of supporting immigration. Compared to surrounding more wealthy suburban towns, Framingham has more social service agencies, more subsided housing, and more group homes. Framingham and the surrounding towns also have a large number of nursing homes and assisted living facilities. The COVID-19 experience at the Framingham Union campus, a community teaching hospital, mirrored the experience of the rest of Massachusetts. MetroWest Medical Center went from two intensive care units to four intensive care units, and nearly half of the admissions during S1 were from nursing facilities. One-quarter of the patients admitted during S1 died. Factors that contributed to this high fatality rate included advanced age and a high rate of comorbidities; a third of the patients had dementia, and a third entered the hospital with a DNR status. Similar to other studies, there was a clear-cut association between poor outcome and advanced age and CCI [12]. The association between poor outcome and obesity was poor, except that obese patients had a higher rate of mechanical ventilation [13,14]. As in other studies, we noted the role of racial disparity in patients admitted with COVID-19 infection [15-18].

In contrast, the death rate during S2 was substantially lower. Although the mean age was similar, the predicted CCI 10-year survival rate was different (21% versus 53%). Most of the admitted patients during S2 were from home, and again, the racial disparity was noted. Obviously, a big difference between the two surges was the treatment given, and most of the patients admitted in December received steroids and antiviral therapy. While it is tempting to assign the improvement in survival and outcomes to these strategies, our observational study cannot prove this.

The future of the COVID-19 pandemic remains uncertain. Months after the second surge in Massachusetts, cases continue to rise, particularly in younger individuals. In other parts of the United States, hospitalization rates are again soaring, fueled by low vaccination rates and perhaps by the emergence of variants. While vaccination remains a cornerstone of the public health measures, better strategies to manage patients admitted with severe COVID-19 pneumonia are needed.

There are limitations to this study that should be considered. This was a retrospective study from medical records review and subject to biases associated with those including selection bias and misclassification or incorrect information on individual charts. This study also included only hospitalized patients, excluding patients that were treated in the ambulatory setting. Lastly, long-term follow-up data on these patients were not obtained. However, despite these limitations, we consider that our studied patients reflect a true representation of the diverse population in Framingham, Massachusetts.

## Conclusions

There was a significant improvement in outcomes particularly in mortality in the second surge in comparison to the first surge. The high fatality rate in the first surge was attributed mainly to advanced age and a high rate of comorbidities. It is worth highlighting that the implementation of treatments such as antiviral therapy occurred after the first surge (May 2020) and may have impacted the better mortality rate in the second surge, yet the patients on the first surge had poorer predicted survival. To this date, better strategies to manage patients admitted with severe COVID-19 pneumonia are still needed.

## References

[1] Holshue ML, DeBolt C, Lindquist S (2020). First case of 2019 novel coronavirus in the United States. N Engl J Med.

[2] Richardson S, Hirsch JS, Narasimhan M (2020). Presenting characteristics, comorbidities, and outcomes among 5700 patients hospitalized with COVID-19 in the New York City area. JAMA.

[3] (2020). Commonwealth of Massachusetts: Man returning from Wuhan, China is first case of 2019 Novel Coronavirus confirmed in Massachusetts. Massachusetts Department of Public Health.

[4] Lipsitz LA, Lujan AM, Dufour A, Abrahams G, Magliozzi H, Herndon L, Dar M (2020). Stemming the tide of COVID-19 infections in Massachusetts nursing homes. J Am Geriatr Soc.

[5] Shen K, Loomer L, Abrams H, Grabowski DC, Gandhi A (2021). Estimates of COVID-19 cases and deaths among nursing home residents not reported in federal data. JAMA Netw Open.

[6] (2021). The R Project for Statistical Computing. https://www.R-project.org/..

[7] Christensen DM, Strange JE, Gislason G, Torp-Pedersen C, Gerds T, Fosbøl E, Phelps M (2020). Charlson Comorbidity Index score and risk of severe outcome and death in Danish COVID-19 patients. J Gen Intern Med.

[8] Inker LA, Astor BC, Fox CH (2014). KDOQI US commentary on the 2012 KDIGO clinical practice guideline for the evaluation and management of CKD. Am J Kidney Dis.

[9] Institute of Medicine (US) Committee on a National Surveillance System for Cardiovascular and Select Chronic Diseases (2011). A nationwide framework for surveillance of cardiovascular and chronic lung diseases.

[10] (2021). Commonwealth of Massachusetts: COVID-19 response reporting. https://www.mass.gov/info-details/covid-19-response-reporting..

[11] Gross JV, Mohren J, Erren TC (2021). COVID-19 and healthcare workers: a rapid systematic review into risks and preventive measures. BMJ Open.

[12] Garg S, Patel K, Pham H (2021). Clinical trends among U.S. adults hospitalized with COVID-19, March to December 2020: a cross-sectional study. Ann Intern Med.

[13] Zhang X, Lewis AM, Moley JR, Brestoff JR (2021). A systematic review and meta-analysis of obesity and COVID-19 outcomes. Sci Rep.

[14] Huang Y, Lu Y, Huang YM, Wang M, Ling W, Sui Y, Zhao HL (2020). Obesity in patients with COVID-19: a systematic review and meta-analysis. Metabolism.

[15] Nau C, Bruxvoort K, Navarro RA (2021). COVID-19 inequities across multiple racial and ethnic groups: results from an integrated health care organization. Ann Intern Med.

[16] Mackey K, Ayers CK, Kondo KK (2021). Racial and ethnic disparities in COVID-19-related infections, hospitalizations, and deaths : a systematic review. Ann Intern Med.

[17] Vahidy FS, Nicolas JC, Meeks JR (2020). Racial and ethnic disparities in SARS-CoV-2 pandemic: analysis of a COVID-19 observational registry for a diverse US metropolitan population. BMJ Open.

[18] Muñoz-Price LS, Nattinger AB, Rivera F (2020). Racial disparities in incidence and outcomes among patients with COVID-19. JAMA Netw Open.

